# A Retrospective Study to Assess the Association Between Binge Drinking and Kidney Diseases

**DOI:** 10.7759/cureus.95967

**Published:** 2025-11-02

**Authors:** Abraham Maramkandathil John, Shristi Kumari, Sumit Sharma, Laveena Sadhnani, Nahla Hakeem, Rajmohan Seetharaman

**Affiliations:** 1 Internal Medicine, Holy Family Hospital, Thodupuzha, IND; 2 Internal Medicine, Anugrah Narayan Magadh Medical College, Gaya, IND; 3 Internal Medicine (Nephrology), Odesa National Medical University, Odesa, UKR; 4 Internal Medicine (Nephrology), Nanjing Medical University, Nanjing, CHN; 5 Internal Medicine, Government Medical College Kannur, Kannur, IND; 6 Pharmacology, Mahatma Gandhi Mission (MGM) Medical College and Hospital, MGM Institute of Health Sciences, Navi Mumbai, IND

**Keywords:** binge drinking, brfss, kidney disease, odds ratio, retrospective study

## Abstract

Introduction: This study delves into the link between binge alcohol drinking and kidney diseases, exploring how overindulgence may erode the body’s vital filtration system.

Aim: To evaluate the association between binge alcohol drinkers and kidney diseases, excluding kidney stones and bladder incontinence, based on demographic and socioeconomic characteristics.

Methods: This retrospective study utilized the Behavioral Risk Factor Surveillance System (BRFSS) database. The disease variable was self-reported kidney diseases, excluding kidney stones, bladder infection, and incontinence, and the risk factor was binge drinking. Control variables included demographic characteristics (age, gender, and race) and socioeconomic factors (education and income). Data were analyzed using cross-tabulations, chi-square tests, and Fisher’s exact tests, with results expressed as odds ratios and 95% confidence intervals.

Results: Binge drinkers are 65% less likely to be associated with kidney diseases than non-binge drinkers. This trend was observed across age groups, with the greatest reduction in kidney disease risk among binge drinkers aged 45-64 years (51% lower likelihood). Male binge drinkers showed a greater reduction (65% lower likelihood) compared to female drinkers (63% lower likelihood). Racial disparities indicated that White binge drinkers had the most significant reduction in kidney diseases (67% lower likelihood). Socioeconomic factors revealed that participants with advanced education and higher incomes showed a 66% and 62% lower likelihood of kidney diseases, respectively, in binge drinkers.

Conclusion: Binge drinking is inversely associated with kidney diseases across demographic and economic groups.

## Introduction

Kidney disease, encompassing both acute and chronic forms, is a significant public health concern that contributes to a substantial burden of illness and healthcare costs worldwide. The kidneys play a vital role in maintaining homeostasis, including fluid balance, blood pressure regulation, and the removal of metabolic waste products. Disruption to these functions can have serious systemic consequences. Among the numerous risk factors for kidney dysfunction, alcohol consumption, particularly binge alcohol drinking, has emerged as an area of growing concern [1,2]. Binge drinking, defined as consuming a large amount of alcohol in a short period (typically five or more drinks for men and four or more for women within two hours), is associated with both immediate and long-term health risks. While some studies have reported neutral or even protective effects of low to moderate alcohol consumption on kidney function, the evidence concerning high-volume episodic drinking is far less reassuring. Binge drinking may induce acute kidney stress by promoting dehydration, raising blood pressure, and increasing oxidative stress, all of which are recognized contributors to renal injury [1,2].

Recent epidemiological and cohort studies have begun to shed light on the complex relationship between alcohol consumption patterns and kidney health [1]. A meta-analysis of prospective studies found that while low to moderate alcohol intake might be associated with a reduced risk of chronic kidney damage, these protective associations were not observed in the context of heavy or binge drinking [1]. Similarly, another dose-response analysis indicated that although alcohol consumption at moderate levels was linked to lower chronic kidney disease (CKD) risk, excessive intake may negate these benefits and increase susceptibility to renal impairment, particularly in vulnerable populations [2].

In population-based research from Korea and Australia, patterns of alcohol consumption were also found to influence kidney-related outcomes. One nationwide study of older adults showed that frequent binge drinking episodes were associated with higher risks of kidney disease, particularly in males [3]. Another cohort study described a U-shaped curve, where moderate drinkers had lower CKD risk, but heavy drinkers faced elevated risks of albuminuria and reduced glomerular filtration rates [4,5].

Given these findings, it is important to examine how binge drinking specifically impacts kidney health in diverse populations. This study aims to investigate the relationship between binge drinking and kidney diseases using US data from the Behavioral Risk Factor Surveillance System (BRFSS) 2022. By exploring the role of alcohol use in the context of demographic and socioeconomic factors, this research seeks to clarify whether binge drinking constitutes a modifiable risk factor for kidney disease in the general adult population.

Aims and objectives

The main goal of this study is to examine the relationship between binge drinking in patients with known kidney dysfunction by utilizing extensive, population-based data from the BRFSS.

The study specifically aims to determine the frequency of binge drinking in different demographic groups and investigate the degree to which indications of renal disease are associated with frequent binge drinking.

The study also seeks to ascertain if excessive drinking is a reliable indicator of declining renal function. The project aims to offer evidence-based insights that can guide clinical practice and assist in the creation of focused public health policies and intervention initiatives by identifying at-risk groups based on behavioral, socioeconomic, and geographic factors.

## Materials and methods

This study is a retrospective cross-sectional analysis conducted using the BRFSS, the largest ongoing, telephone-based health survey system in the United States. The BRFSS collects data annually from non-institutionalized adults aged 18 years and above across all 50 states, the District of Columbia, and U.S. territories. The database is de-identified and publicly available; therefore, institutional review board (IRB) approval was not required [6].

Data collection and variables

Data were extracted using the BRFSS Web Enabled Analysis Tool (WEAT) for the year 2022. The disease variable in this study was kidney disease, excluding conditions such as kidney stones, bladder infections, or incontinence, while the risk factor variable was binge drinking. Control variables included demographic and socio-economic characteristics. Demographic characteristics were age (_AGE_G), categorized into four groups (18-24, 25-44, 45-64, and 65+); gender (SEX), categorized as male and female; and race (_RACEGR), categorized as White (non-Hispanic), Black (non-Hispanic), and non-Hispanic/other races. Socio-economic characteristics included education level (EDUCA), categorized as basic education (high school or less) and advanced education (some college or higher), and annual income (_INCOMG), categorized as <$50,000 and >$50,000. Participants who responded to the selected disease, risk factor, and control variables were included in the study, ensuring completeness of data for analysis [7].

Statistical analysis

Descriptive data in the form of numbers and percentages were generated for each variable using cross-tabulations in the WEAT in the BRFFS. Data were stored in Microsoft Excel (Microsoft Corporation, Redmond, WA), and statistical analysis was performed using R version 4.3.1 (R Foundation for Statistical Computing, Vienna, Austria). Odds ratios (ORs) with 95% confidence intervals (CIs) were calculated to estimate the association between binge drinking and kidney diseases [8].

## Results

In the year 2022, in the United States of America, 392,529 people participated in the BRFSS study. Out of the total 392,529 participants who reported binge drinking, 1059 (1.9%) had kidney disease. In contrast, among 17,287 participants who did not report binge drinking, 5.1% had kidney disease.

The odds ratio of 0.35 indicates that participants who reported binge drinking were 65% less likely to have kidney disease compared to those who are not binge drinkers, and this is a significant association, as shown in Table 1.

**Table 1:** Prevalence of binge drinking in participants with self-reported kidney disease. * Significant.

Binge drinking	Yes	Kidney disease		OR (CI)
		Yes	No	0.35* (0.3284, 0.3726)
		1059 (1.9%)	55747 (98.1%)	
	No	17287 (5.1%)	318436 (94.9%)	

The prevalence of kidney disease in participants who were binge drinkers was 57 (0.9%) in the 18-24 years age group, 220 (1%) in the 25-44 years age group, 409 (2.1%) in the 45-64 years age group, and 373 (4.8%) in participants aged 65 years and above. When gender was considered, kidney disease was more prevalent in females who were binge drinkers at 444 (2%), as compared to males who indulged in binge drinking at 615 (1.8%). Among racial groups, White, non-Hispanic participants who were binge drinkers had a prevalence of 740 (1.8%), Black, non-Hispanic participants who indulged in binge drinking had a prevalence of 12,954 (5.3%), and Hispanic/other groups had a prevalence of 198 (1.9%).

The likelihood of an association between kidney disease and binge drinking, expressed as odds ratio, was 27.6% (OR: 1.276) in 18-24 years, 39.6% (OR: 0.604) in 25-44 years, 51.9% (OR: 0.481%) in 45-64 years, and 45% (OR: 0.55) in participants aged 65 years and above. When considering gender, the likelihood of an association between kidney disease in male participants who indulged in binge drinking was 65.1% (OR: 0.349), and in females who indulged in binge drinking was 63.8% (OR: 0.362). Among different races, the likelihood of the association of kidney disease among White, non-Hispanics who were binge drinkers was 67.6% (OR: 0.324), among Black, non-Hispanics was 60% (OR: 0.4), and among Hispanic/others was 55.8% (OR: 0.442), as represented in Table 2.

**Table 2:** Prevalence of binge drinking in participants with self-reported kidney disease based on demographic characteristics. * Significant.

	Binge drinking	N	Participants with kidney disease	Participants without kidney disease	OR (CI)
Age groups					
18-24 years	Yes	6144	57 (0.9%)	6087 (99.1%)	1.276 (0.916, 1.7581)
	No	17705	129 (0.7%)	17576 (99.3%)	
25-44 years	Yes	23076	220 (1%)	22856 (99%)	0.604* (0.5197, 0.699)
	No	70807	1111 (1.6%)	69696 (98.4%)	
45-64 years	Yes	19764	409 (2.1%)	19355 (97.9%)	0.481* (0.4332, 0.5329)
	No	110823	4664 (4.2%)	106159 (95.8%)	
65+ years	Yes	7822	373 (4.8%)	7449 (95.2%)	0.55* (0.4934, 0.6114)
	No	136388	11383 (8.3%)	125005 (91.7%)	
Gender					
Male	Yes	34481	615 (1.8%)	33866 (98.2%)	0.349* (0.3205, 0.379)
	No	150024	7425 (4.9%)	142599 (95.1%)	
Female	Yes	22325	444 (2%)	21881 (98%)	0.362* (0.3279, 0.3984)
	No	185699	9862 (5.3%)	175837 (94.7%)	
Race					
White, non-Hispanic	Yes	41706	740 (1.8%)	40966 (98.2%)	0.324* (0.3003, 0.3493)
	No	245338	12954 (5.3%)	232384 (94.7%)	
Black, non-Hispanic	Yes	3275	83 (2.5%)	3192 (97.5%)	0.4* (0.316, 0.5008)
	No	26337	1607 (6.1%)	24730 (93.9%)	
Hispanic/others	Yes	10526	198 (1.9%)	10328 (98.1%)	0.442* (0.3801, 0.5127)
	No	54515	2264 (4.2%)	52251 (95.8%)	

Table 3 shows that the prevalence of kidney disease in participants who indulged in binge drinking was 382 (2.4%) with basic education and 676 (1.7%) in those with advanced education. When income was considered, the prevalence of kidney disease in binge drinking participants with an annual income of <$50,000 was 448 (2.8%), and 483 (1.4%) in participants with an income of >$50,000.

**Table 3:** Prevalence of binge drinking in participants with self-reported kidney disease based on education level and socio-economic characteristics. * Significant.

	Binge drinking	N	Participants with kidney disease	Participants without kidney disease	OR (CI)
Education level					
Basic education	Yes	16162	382 (2.4%)	15780 (97.6%)	0.391* (0.3508, 0.434)
	No	99757	5821 (5.8%)	93936 (94.2%)	
Advanced education	Yes	40498	676 (1.7%)	39822 (98.3%)	0.332* (0.3067, 0.3592)
	No	234727	11414 (4.9%)	223313 (95.1%)	
Annual income ($)					
<50,000	Yes	15892	448 (2.8%)	15444 (97.2%)	0.384* (0.3477, 0.4228)
	No	116628	8195 (7%)	108433 (93%)	
>50,000	Yes	34069	483 (1.4%)	33586 (98.6%)	0.373* (0.3387, 0.4093)
	No	156058	5798 (3.7%)	150260 (96.3%)	

The likelihood of the association between kidney disease and binge drinking, expressed as odds ratio, was 60.9% (OR: 0.391) in participants with basic education and 66.8% (OR: 0.332) in those with advanced education. When income was considered, the likelihood of the association between kidney disease and binge drinking was 61.6% (OR: 0.384) in participants with an annual income of <$50,000 and 62.7% (OR: 0.373) in those with >$50,000 annual income.

## Discussion

This retrospective study explored the association between binge drinking and kidney diseases, excluding conditions such as kidney stones, bladder infections, or incontinence, using the 2022 BRFSS dataset. Contrary to conventional expectations, findings indicated an inverse relationship between binge drinking and kidney disease prevalence across various demographic groups. Individuals who reported binge drinking exhibited a 65% lower likelihood of having kidney disease compared to non-binge drinkers. This protective association was particularly marked among those aged 45-64 years, who experienced a 51.9% lower risk, and among male binge drinkers, whose risk was reduced by 65.1%. Although a higher prevalence of kidney disease was observed in the age group of 18-24 years, the difference was not statistically significant. The analysis also revealed disparities based on race, with White binge drinkers showing the most substantial reduction in disease likelihood (67.6%), followed by Black individuals (60%) and those from non-Hispanic or other racial backgrounds (55.8%). Furthermore, participants with higher education and income levels demonstrated lower odds of kidney disease (66.8% and 62.7%, respectively) if they engaged in binge drinking. These findings suggest that the interplay between alcohol use and kidney disease is influenced not only by consumption patterns but also by socio-demographic context.

While alcohol is commonly associated with adverse renal outcomes, including CKD and acute kidney injury [1,9-15], the apparent protective effect of binge drinking observed here challenges that narrative. Moeinzadeh et al. [10], for example, reported that occasional alcohol intake was linked to significantly greater odds of advancing to stages 3 or 4 CKD compared to stage 1. In contrast, our data demonstrated a 65% lower likelihood of kidney disease among binge drinkers. This aligns with findings from Cheungpasitporn et al. [13], as well as meta-analyses by Li et al. [1] and Lee et al. [14], which also identified a potential protective association between moderate to high alcohol consumption and CKD in males. Similarly, large-scale studies by Lai et al. [16], Koning et al. [17], and Reynolds et al. [18], along with recent Taiwan Biobank data [19] and a review by Zhan et al. [9], support an inverse association between alcohol use and CKD risk in some populations.

However, other studies raise caution. Tanaka et al. [15] reported that frequent, high-volume alcohol intake was associated with an increased incidence of proteinuria and significant reductions in estimated glomerular filtration rate (eGFR), particularly among women. Shibata et al. [20] similarly suggested that high per-drinking-day alcohol consumption may lead to glomerular hyperfiltration, a potential early indicator of kidney damage. These conflicting results underscore the complexity of the relationship between alcohol use and renal function, and the possibility that gender, frequency, and volume of consumption all modulate risk [1,10-20].

Age, gender, and ethnicity further shaped the observed outcomes. Among older adults (65+), binge drinkers showed a 45% reduction in kidney disease risk. Similarly, both female (63.8%) and male (65.1%) binge drinkers exhibited a considerably lower risk compared to non-binge drinkers. Racially, the greatest decrease was again observed among White participants, followed by Black and non-Hispanic/other individuals.

Importantly, while our findings suggest a negative association between binge drinking and kidney disease, they do not imply that binge drinking is protective or recommended. Prior research has shown that excessive alcohol use, especially when sustained, can increase the risk of end-stage renal disease [12,18]. The apparent protective effects in certain populations may reflect confounding factors or selection bias rather than a true causal relationship.

Ultimately, these findings highlight the need for more nuanced, longitudinal research to disentangle the complex relationship between alcohol use, especially binge patterns, and kidney health across diverse populations [16-20].

Limitations

This study has several limitations. First, the BRFSS dataset is based on telephone surveys, which may exclude individuals without access to landlines or those who do not use them, potentially leading to underrepresentation of certain populations. Second, the study relies on self-reported data rather than clinical histories or examinations, which introduces the possibility of recall bias. Furthermore, the dataset lacks detailed information regarding the type, severity, or timing of kidney disease diagnoses, which could impact the associations observed. Lastly, as a retrospective observational study, this research identifies associations but does not establish causality. Prospective studies, such as those referenced in Lai et al. [16], Koning et al. [17], and Shibata et al. [20], are necessary to clarify potential causal pathways.

## Conclusions

This study demonstrated a reduced likelihood of kidney disease among binge drinkers across various demographic and socio-economic groups. The reduction was particularly pronounced among older adults, females, White participants, and individuals with higher levels of education and income. Future research should prioritize prospective studies to explore the underlying mechanisms of these associations and assess whether binge drinking, along with other confounding factors, contributes to the observed outcomes. Such studies would be instrumental in developing targeted guidelines and preventive strategies for kidney diseases, taking into account lifestyle and socio-demographic factors.
